# The Problem of Hospital Malnutrition in the African Continent

**DOI:** 10.3390/nu11092028

**Published:** 2019-08-30

**Authors:** Renée Blaauw, Esther Achar, Robin C Dolman, Janetta Harbron, Merel Moens, Faith Munyi, Dzifa Nyatefe, Janicke Visser

**Affiliations:** 1 Division of Human Nutrition, Faculty of Medicine and Health Sciences, Stellenbosch University, Cape Town 8000, South Africa; 2 Centre of Excellence for Nutrition, North-West University, Potchefstroom Campus, Potchefstroom 2520, South Africa; 3 Division of Human Nutrition, Faculty of Health Sciences, University of Cape Town, Cape Town 8000, South Africa

**Keywords:** hospital malnutrition, adults, African continent

## Abstract

This study aims to determine the prevalence of risk of malnutrition on admission and discharge in African hospitals, and to identify the association with selected indicators. In this multi-center prospective cohort study, adult patients from hospitals in South Africa, Kenya, and Ghana were screened on admission and discharge and contacted 3 months post-discharge. Relevant morbidity and mortality outcomes were assessed. At risk of malnutrition was indicated if NRS-2002 score ≥3. Adult patients (*n* = 2126; 43.11 years, IQR: 31.95–55.60; 52.2% female) were screened on admission and 61% were identified as at risk of malnutrition. The proportion of at-risk patients for the three hospitals in Kenya and Ghana (66.2%) were significantly higher than that of the three South African hospitals (53.7%) (Chi^2^ = 31.0; *p* < 0.001). Discharge risk of malnutrition was 71.2% (*n* = 394). Mean length of stay (LOS) was 6.46 ± 5.63 days. During hospitalization, 20.6% lost ≥5% body weight, 18.8% were referred for nutrition support, and discharge BMI (23.87 ± 7.38 kg/m^2^) was significantly lower than admission BMI (24.3 ± 7.3 kg/m^2^) (*p* < 0.001). Admission nutrition risk was associated with lower admission and discharge BMI (*p* < 0.001), longer LOS (*p* < 0.001), increased 3-month re-admission rates (Chi^2^ = 1.35; *p* = 0.245) and increased mortality (Chi^2^ = 21.68; *p* < 0.001). Nearly two-thirds of patients were at risk of malnutrition on admission. This was associated with longer LOS and greater hospital mortality. The nutritional status of patients deteriorated during hospitalization. Routine screening practices with appropriate nutrition support action should be implemented as a matter of urgency.

## 1. Introduction

Globally, the prevalence of malnutrition in adults on admission to hospital varies between 11–74% [1,2,3,4,5,6,7,8,9,10,11,12,13,14]. In Africa, nutritional screening is not routinely performed and reliable statistics indicating the extent of the problem are not available. Knowing the prevalence of malnutrition and identifying at-risk patients timeously should thus be a priority since it is well-known that malnutrition is associated with increased hospital-related complications and infections (morbidity) [8,11,15,16], longer length of stay (LOS) [1,2,8,9,10,11,15,16,17,18], increased costs related to treatment [1,10,11,18,19] and higher mortality [3,5,9,10,11,15,18,19]. Post-discharge, malnourished patients are more frequently re-admitted, and present with higher morbidity and mortality rates [5,10,18]. However, effective screening and referral of at-risk patients and timely implementation of nutritional support have the opposite effects and improve clinical outcomes [20,21]. Various screening tools are available to identify patients at nutritional risk. The NRS-2002 tool has been shown to accurately identify patients at risk of becoming malnourished and in need of nutrition support [4,16,22]. A total of 11 screening tools were evaluated for their ability to detect malnutrition in patients from acute care and hospital-based ambulatory care settings. The NRS-2002 tool was the only tool to receive a grade 1 recommendation [23] and was thus utilized in the present study. Therefore, this study aims to determine the prevalence of risk of malnutrition on admission and discharge and to identify the association thereof with select in-hospital and post-discharge indicators.

## 2. Methods

### 2.1. Participants and Study Design

In this multi-country, multi-center prospective cohort study, adult patients (≥18 years) admitted to hospital within the preceding 48 h and who gave informed consent were screened for malnutrition on admission and discharge, and contacted 3 months post-discharge. The following hospitals were conveniently selected and participated: three hospitals from South Africa (Tygerberg Academic Hospital, Cape Town; Groote Schuur Academic Hospital, Cape Town; Chris Hani Baragwanath Hospital, Johannesburg), two hospitals from Kenya (Aga Khan University Hospital, Nairobi; Mbagathi District Hospital, Nairobi), and one hospital from Ghana (Korle Bu Teaching Hospital, Accra). The following patient-groups were excluded: pediatric patients (<18 years); pregnant and lactating females, patients admitted to an intensive care unit (ICU), burns or relevant acute care wards; patients admitted to psychiatry or eating disorder units; as well as patients on dialysis.

The sample size calculation was based on the primary objective, and to determine the proportion of patients at risk of malnutrition on admission to hospital with a precision of 3.6%, 90% power and a 95% confidence interval. To achieve this, 2100 participants (350 per hospital) were required.

### 2.2. Measurements

Facility-coordinators and fieldworkers were responsible for data gathering at each of the facilities. Extensive fieldworker training and adherence to standard operating procedures ensured quality of data. Baseline data were collected within the first 48 h of hospital admission. Patients that were admitted for ≥7 days qualified for inclusion in the discharge group. This data was then obtained at either actual discharge or day 28 of hospitalization for those staying longer.

### 2.3. Malnutrition Risk Screening Tool

Nutritional risk was evaluated on admission and discharge using the Nutritional Risk Screening-2002^®^ (NRS-2002) scoring system developed by the European Society for Clinical Nutrition and Metabolism (ESPEN) [24]. This tool takes into consideration body mass index (BMI), unintentional weight loss, disease severity, decreased food intake/loss of appetite, and age and has been shown to accurately identify those at risk of malnutrition. At risk of malnutrition was indicated if NRS-2002 scored ≥3 [24].

### 2.4. Other Data Collected

Weight and height assessments were done on admission and weekly (weight) until discharge. Patients were also asked about usual weight and weight changes in the period up to 6 months before admission. Body mass index was calculated after correcting for edema and interpreted according to the established guidelines [25]. The percentage weight loss was calculated by subtracting current weight from usual weight and expressed as a percentage (a negative value indicates weight gain).

Data relating to food intake or loss of appetite prior to admission, as well as a reflection of actual food intake during hospitalization (decreased intakes recorded as ¾, ½, or ¼ plates) was obtained. Whether the patient was referred for nutritional support during their hospital admission was also noted.

Relevant medical data including hospital admission and discharge dates, gender, reasons for hospitalization, medical diagnosis, development of in-hospital complications requiring medical intervention [26], and mortality were noted.

At 3 months post-discharge, a sub-sample of patients were telephoned to assess their self-reported progress, including any hospital readmissions, conditions requiring medical intervention, weight changes, level of appetite, and mortality (if relevant) in the past 3 months.

### 2.5. Data Analysis

MS Excel and STATISTICA (version 13.4, 2018) was used to capture and analyze the data, respectively. Summary statistics are used to describe the variables. Distributions of nominal variables are presented as frequency tables. Medians and means are used as the measures of central location for ordinal and continuous responses and standard deviations and interquartile ranges (IQR) as indicators of spread. Results are reported as means ± standard deviation (SD). Relationships between two continuous variables were analyzed with the Pearson or Spearman correlation, if the continuous variables were not normally distributed. The relationships between continuous dependent variables and nominal independent variables (factors) were analyzed using one-way ANOVA, if the influence of one factor on dependent variables were measured and two-way factorial ANOVA when the influence of two or more factors were measured. When nominal dependent variables were compared versus nominal independent variables with contingency tables, the associations are reported with maximum likelihood chi-square tests. Statistical significance of 5% were used in all hypothesis tests. The relevant sample size for each of the variables discussed are listed in brackets.

### 2.6. Ethical Principles

The Health Research Ethics Committee, Stellenbosch University approved the study in October 2014 (N14/06/061). Thereafter, it was submitted and approved by local ethics committees and regulatory bodies in the respective countries and relevant hospitals. The study was carried out following the rules of the Declaration of Helsinki of 1975, revised in 2013. All participants provided voluntary informed consent before participating in the study and could withdraw at any time.

## 3. Results

A total of 2126 patients were included and analyzed on admission. Of these, 557 cases were analyzed at discharge and post-discharge data were collected for 452 patients.

### 3.1. Admission Data

The median age of patients included was 43.11 years (IQR: 31.95–55.60 years), with 52.2% being female. Distribution of patients according to gender, country, and primary diagnosis are presented in Table 1.

Median BMI of patients was 23.7 kg/m^2^ [IQR: 19.7–28.7 kg/m^2^]. However, just under one-fifth (*n* = 356, 16.7%) of patients presented with a BMI <18.5 kg/m^2^. Females had a significantly higher BMI (females: 26.26 ± 7.88 vs. males: 23.04 ± 5.66, *p* < 0.001). Significantly more females also presented with obesity than males (*p* < 0.001) (Table 2).

In total, 1153 (54.3%) patients experienced weight loss before hospital admission, with an average percentage weight loss of 6.77 ± 10.81% (−26.32% to 61.54% range). Just under a quarter experienced weight loss exceeding 5% in the period before hospitalization (512/2125, 24%) (Table 3). Significant differences were found between the primary diagnostic groups and the percentage weight loss prior to admission (*p* < 0.001). The greatest weight loss was experienced by patients with HIV/TB (14.06%), oncology (10.87%), and endocrine diseases (8.06%).

The average NRS-2002 score was 2.65 ± 1.63. A total of 1714 (80.6%) patients succeeded to the final assessment phase of the NRS-2002. Of these, 75.1% (1288/1714) scored positively for being at risk of malnutrition (NRS ≥ 3), which represents 60.6% (1288/2125) of all patients on admission (Table 3).

Despite the large proportion of patients being at risk of malnutrition on admission to hospital, a mere 8.09% (*n* = 172/2126) of the admitted patients were referred for nutritional support within the first 48 h after admission (Table 3). This represented 13.4% (172/1288) of all at-risk patients.

### 3.2. Discharge Data:

Mean hospital LOS was 6.46 ± 5.63 days and this was positively, albeit weakly, related to age (r = 0.097, *p* < 0.001) and percentage weight loss prior to admission (r = 0.137, *p* = 0.002). During hospitalization, 58.6% (*n* = 313/534) of patients lost weight, with 20.6% (110/534) losing more than 5% body weight (Table 3). Just over half (54.4%, *n* = 295) of the patients reported a reduced food intake (≤75% of usual intake) while in hospital and only 18.8% (*n* = 102/544) of all patients were referred for specialized nutritional support during hospitalization.

Just less than half (40.26%, *n* = 215) of the patients developed complications requiring medical intervention during hospitalization. Patients developed an average of 0.79 ± 1.19 (0–5 range) complications. Significant differences were found between primary diagnosis and the number of complications incurred during hospitalization (*p* < 0.001), with the highest number of complications found among patients with HIV/TB (1.96), general surgical conditions (1.22), and gastroenterology conditions (1.31).

The average NRS score was 3.11 ± 1.46 at discharge and the proportion of patients at risk of malnutrition was 71.2% (*n* = 394/553) (Table 3).

### 3.3. Post-Discharge Data

In total, 452 patients were available for follow-up telephonically 3 months after the hospital discharge. Self-reported weight changes revealed that the majority (197/381, 51.7%) had gained weight post-discharge, while the weight of 21.5% (82/381) remained constant, and 26.8% (102/381) had lost weight in the post-discharge period.

Of the group, 73 (36.86%) developed a complication that required medical intervention and 66 (17%) had been readmitted to hospital in that period. Of those readmitted, the percentage weight loss prior to baseline admission was significantly higher (12.35 ± 15.94%) than the weight loss in those not readmitted (6.44 ± 8.78%) (*p* = 0.001). BMI on primary admission was negatively related to the number of complications that developed in the follow-up period (r = −0.229, *p* = 0.001).

In total, 63 (14%) patients died in this 3-month period, which was more than the 36 (1.7%) patients that died during hospitalization.

### 3.4. Changes in Nutritional Risk from Admission to Discharge (Paired Data)

Wilcoxon signed rank testing for non-parametric paired data revealed that the NRS score on admission (2.95 ± 1.15) was significantly lower than the discharge score (3.11 ± 1.46) (*p* < 0.001), whereas the admission average BMI (24.31 ± 7.31 kg/m^2^) was significantly higher than the discharge BMI (23.87 ± 7.38 kg/m^2^) (*p* < 0.001). The inverse link between NRS score and BMI was confirmed with a significant negative relationship noted between admission NRS score and a lower BMI (r = −0.302, *p* = 0.001). It was also noted that the proportion of patients with a BMI <18.5 kg/m^2^ at discharge (24.3%) was significantly more than the admission values (19.17%, *p* = 0.003), indicating a shift from a normal to decreased BMI status that developed during hospitalization.

### 3.5. Associations between At risk of Malnutrition and Various Parameters

Being nutritionally at risk of malnutrition on admission to hospital was associated with various parameters, including a lower admission and discharge BMI (*p* < 0.001), longer LOS (*p* = 0.001), and increased mortality (*p* < 0.001). (Table 4).

**Figure 1:**
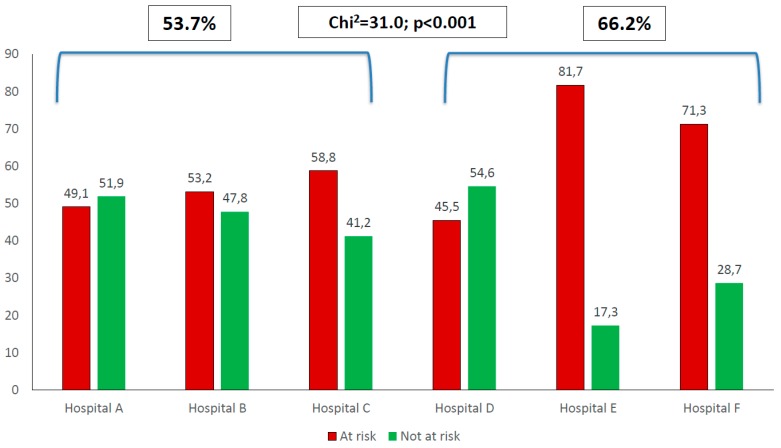
Proportion of patients at risk of malnutrition on admission per hospital.

Significant differences were found for at risk of malnutrition between the six hospitals (Chi^2^ = 164.6, *p* < 0.001). When the three hospitals from South Africa (Hospitals A–C) were compared to the three hospitals from outside of South Africa (Hospitals D–F), the proportion of at-risk patients in the former (53.7%) were significantly less than the proportion of at-risk patients in the hospital outside of South Africa (66.2%) (Chi^2^ = 31.0, *p* < 0.001). (Figure 1.)

**Figure 2:**
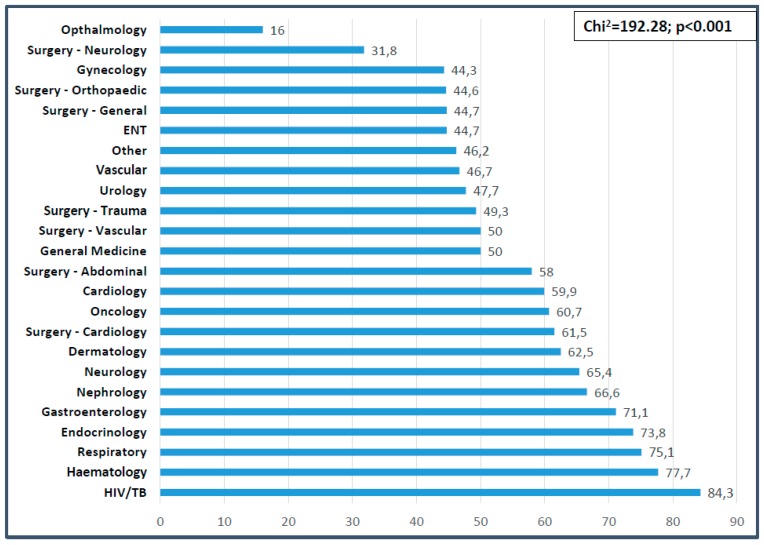
Proportion of patients at risk of malnutrition per diagnostic category at baseline.

Significant differences were also found between the NRS score obtained and diagnostic category (more patients at risk of malnutrition in medical versus surgical or oncology patients, *p* < 0.001). Significant differences were noted between the proportion of patients at risk of malnutrition and the primary diagnosis (Chi^2^ = 192.28, *p* < 0.001). The highest prevalence of malnutrition risk was found in patients with HIV/TB (84.3%), hematology (77.7%), respiratory (75.1%), endocrinology (73.8%), and gastroenterology (71.1%) conditions (Figure 2).

## 4. Discussion

Hospital malnutrition is known to be associated with adverse outcomes. This study set out to determine the prevalence of at risk of malnutrition adults on admission to and discharge from hospital and to determine relevant associations with various indicators. To the authors’ knowledge, this is the first multi-country study of this nature and size conducted on the African continent, producing much needed hospital malnutrition prevalence and related data in this context.

Although a wide age-range was included, the median age of the patients (43 years) was at least a decade younger than those reported for similar studies done in Europe, ranging between 57–72 years [1,2,8,15,20,21]. It is closer to the mean ages of similar studies conducted on the African continent which reported a mean age of 34 years (Burundi) [27], 37 years (Uganda) [3], 41 years (Zambia) [28], 47 years (Cameroon) [29], and 48 years (South Africa) [6], highlighting the differences in patient profiles between continents.

### 4.1. On Admission

A normal mean BMI (24.7 kg/m^2^) was found, but the range was quite varied (8–60 kg/m^2^). Females presented with a significantly higher BMI than males, a finding that was also reported for another South African study [6] and in line with local population statistics for South Africa [30], and Ghana and Kenya [31].

More than half the study participants (54%) experienced weight loss prior to admission and in a quarter of cases, the weight loss exceeded 5%. Sorenson et al. [15] reported that 20.4% of patients lost more than 5% of body weight prior to admission and other groups reported that 16.2% [22] and 42% [29] of patients lost more than 10% of body weight prior to admission. As expected, the biggest percentage of weight loss was associated with chronic disease conditions, i.e., infectious diseases (HIV/TB), oncology and endocrine diseases.

Sixty percent of all patients in this study scored positively for being at risk of malnutrition (NRS score ≥ 3) on admission. These results are in line with another study (Switzerland) using the same tool, that identified 62.7% [19] of patients to be at risk of malnutrition. Most of the other studies reported significantly lower prevalence, ranging from 14–38% [1,4,5,8,9,13,14,15,16,18,22,32,33]. Taken together, the prevalence ranged from 14–63% using the NRS-2002 tool in hospitals from mostly Europe, one study from Brazil, and the current data from Africa.

The proportion of at-risk patients for the three hospitals in Kenya and Ghana (66.2%) were significantly higher than that of the three South African hospitals (53.7%). Using a different tool (MUST), another study from South Africa reported a risk of malnutrition of 72.3% [6]. Data from Zambia [28] reported a 59.7% and Uganda [3] a 25–59% prevalence. Hospital malnutrition (defined as ≥10% weight loss from usual weight) was reported to be 47.3% in Burundi [27] and data from Cameroon (defining malnutrition as BMI < 18.5 kg/m^2^, and/or MUAC < 22 cm for women and 23 cm for men) reported 19.3% [29]. It is clear that there is a problem with hospital malnutrition on the African continent.

In the present study, the largest proportion of at-risk patients were seen in those with chronic diseases, with HIV/TB patients presenting with the highest prevalence at 84%. Bearing in mind the persistent malnutrition (undernutrition) reported on the African continent at 20% [34] and the unique additional disease burden in the form of HIV/AIDS and tuberculosis (independently associated with malnutrition) [35], the findings are not unexpected. Furthermore, Niyongabe et al. [27] reported that with an increase of HIV seropositive and AIDS cases (hospitalized patients in East Africa), the nutritional status decreased. Although wasting was the initial nutritional complication of HIV/AIDS to be recognized, and lipodystrophy followed the initial application of HAART, it should be noted that sarcopenia and frailty may be some of the most important challenges in the near future that will need specialized nutrition and other support in this context [36]. Following infectious diseases, hematological, respiratory, endocrine, and oncology patients presented with the highest nutritionally at-risk proportions in our study. Corresponding prevalence rates associated with respiratory diseases range from 42–67% [10,15,19,33]. Lower prevalence rates for endocrinology have been reported with 30% [1] and 48% [10]. However, the prevalence rates for patients associated with oncology range from as low as 34% [1] and 44% [33] to values exceeding those found in the present study, at 70% [19] and 71% [10]. These data identify conditions falling in the “medical” category to be associated with risk of malnutrition.

### 4.2. During Hospitalization and at Discharge

More than half (59%) of the patients lost further weight during hospitalization and in 20% of cases, the weight loss exceeded 5% of body weight. This resulted in a significantly lower discharge mean BMI, compared to admission values and a greater proportion of patients with BMI values below 18.5 kg/m^2^ (paired data). Alvarez-Hernandez [1] also reported significantly lower discharge BMI versus admission values with 39% of patients losing weight while in hospital. At discharge, 71% of patients in the present study were classified as at-risk of malnutrition, which was significantly more than patients at-risk on admission. Another group reported similar findings [1].

Factors contributing to the worsening of nutritional status during hospitalization could be inadequate food intake and inappropriately low referrals for nutritional support. Food intake during hospitalization was poor with 54% of patients reporting a reduced intake. This was substantially more than the 32.4% of hospitalized patients with a reduced food intake reported by Sorensen et al. [15]. During hospitalization, 19% of patients were referred for nutrition support and fewer (17%) actually received any nutrition support. This was much lower than the nutrition support provided to 27.7% [1], 40.3% [19], and 57.6% [12] of malnourished patients in other studies. It is clear from the literature that provision of nutrition support to at-risk patients remains a challenge.

### 4.3. Post Discharge

Self-reported information was obtained telephonically 3-months post-discharge. Hospital admission BMI showed significant negative correlations with the number of complications requiring medical interventions in the 3-month period. Again, this demonstrates the greater susceptibility of undernourished individuals to develop complications.

Hospital readmission was noted in 17% of cases. A greater weight loss prior to initial hospital admission and a lower BMI on admission were found in those readmitted. The positive association between poor nutritional status and hospital re-admission has also been confirmed by Koren-Hakim et al. who reported a 46.5% readmission rate (6-months post-discharge) positively associated with the at-risk group (using MNA-SF tool) [5]. Mortality rates in the 3-months post-discharge period were higher than during hospitalization. Other groups reported similar findings with more deaths occurring post-discharge [3,5].

### 4.4. Associations with Malnutrition

Being at risk of malnutrition on admission to hospital was associated with various parameters including lower BMI on admission and discharge, longer LOS and higher mortality. Although more complications developed during hospitalization and post-discharge in the at-risk group, and the latter patients were readmitted more, this did not differ significantly from the non-at-risk group.

As found in the present study, LOS was positively associated with at-risk of malnutrition by various groups [1,2,4,8,9,10,11,15,16,18,37,38]. However, others did not find this association [5,19,29]. Our finding of an association between at-risk and increased complications [8,11,15,16], more readmissions [5,10,18,37,38] and greater mortality [5,9,10,11,18,19,27,37,38] was also reported by others.

The large economic implications of malnutrition in the hospital setting because of longer LOS, more complications, and readmissions has been displayed in other countries [1,10,11,19,38], but not in the African continent as yet.

## 5. Limitations

Patients had to stay in the hospital for seven days or longer to be included in the discharge group. With an average length of stay of 6 days, the study sample for the discharge data and ultimately for the post-discharge data, was smaller than anticipated. This resulted in loss of statistical power to detect significant associations for paired data. Missing data were not imputed resulting in various group sizes. It is possible that selection bias was introduced if the sicker patients did not consent to participate. Because of ethical restriction on data access, it was not possible to obtain information from those that did not consent. Self-reported data were used for some of the variables which could introduce reporting bias.

## 6. Conclusions

Nearly two-thirds of all patients were found to be at-risk of malnutrition on admission, placing African patients at the top of the range in relation to the global statistics. This was associated with longer length of stay and greater hospital mortality. The nutritional status of patients deteriorated during hospitalization. The reduced food intake and poor referral practices for nutrition support could have contributed to this. Routine screening practices with appropriate specialized nutrition support action should be prioritized and implemented as a matter of urgency in African hospitals. There is a need for more studies in this area across Africa.

## Figures and Tables

**Table 1:** Distribution of patients on admission according to gender, country, and primary diagnosis.

Variables	*n*	Percentage (%)
**Gender** (*n* = 2126)		
Male	1016	47.8
Female	1110	52.2
**Country** (*n* = 2126)		
South Africa	927	43.6
Kenya	797	37.5
Ghana	402	18.9
**Primary diagnosis** (*n* = 2126)		
Cardiology	162	7.6
Endocrine	80	3.8
Gastroenterology	173	8.1
General medicine	106	5.0
Gynecology	140	6.6
Hematology	99	4.7
HIV/TB	229	10.8
Medical Other *	99	4.7
Nephrology	45	2.1
Neurology	81	3.8
Oncology	173	8.1
Respiratory	133	6.3
Surgery, Abdominal and Trauma	218	10.3
Surgery, Other **	344	16.2
Urology	44	2.1

* Medical Other includes: dermatology, ear, nose and throat, ophthalmology, vascular. ** Surgery Other includes: cardiac, orthopedic, vascular, general. Abbreviations: HIV: human immunodeficiency virus; TB: tuberculosis.

**Table 2:** Body mass index classification by gender on admission.

Variables	Unit	Whole Group (*n* = 2123)	Males (*n* = 1016)	Females (*n* = 1107)
**BMI, corrected** (kg/m^2^)				
On admission	Mean ± SD	24.7 ± 7.1	23.0 ± 5.66	26.26 ± 7.88 *
	Median	23.7	22.2	25.42
	IQR	19.72–28.76	19.13–26.04	20.63–31.0
**BMI, categories** (kg/m^2^)	*n*, (%)			
<18.5 kg/m^2^		356 (16.7)	188 (18.5)	168 (15.2)
18.5–24.9 kg/m^2^		863 (40.6)	515 (50.7)	348 (31.4)
25.0–29.9 kg/m^2^		468 (22.0)	204 (20.1)	264 (23.8)
30.0–39.9 kg/m^2^		358 (16.8)	95 (9.4)	263 (23.8) **
≥40.0 kg/m^2^		78 (3.7)	13 (1.3)	65 (5.9)

Data are presented as *n* (%), mean ± SD (median) and IQR. * Significantly different compared to males (*p* < 0.001). ** Significantly different compared to male overweight/obese group (*p* < 0.001). Abbreviations: BMI: body mass index; IQR: interquartile range.

**Table 3:** Nutritional variables on admission versus discharge.

Variables			
	Unit	Mean ± SD	Range (Minimum–Maximum)
**BMI, corrected**	kg/m^2^		
On admission (*n* = 2123)		24.7 ± 7.1	7.9–59.7
At discharge (*n* = 553)		23.87 ± 7.38 *	10.5–51.77
**% weight loss**	%		
On admission (*n* = 1153)		6.8 ± 10.8	−26.3–61.54
At discharge (*n* = 553)		1.67 ± 7.85	−33.3–41.8
**NRS score**			
On admission (*n* = 2125)		2.65 ± 1.63	0–6
At discharge (*n* = 525)		3.11 ± 1.46	0–6
		*n* (%)	
**Weight loss**			
On admission ^1^ (*n* =2 125)		1153 (54.3)	
At discharge ^2^ (*n* = 554)		313 (58.6)	
**>5% weight loss**			
On admission (*n* = 2125)		512 (24.1)	
At discharge (*n* = 553)		110 (20.6)	
**BMI <18.5 kg/m^2^**			
On admission (*n* = 2123)		356 (16.7)	
At discharge (*n* = 532)		129 (24.3)	
**At nutritional risk (NRS ≥ 3)**			
On admission (*n* = 2125)		1288 (60.6)	
At discharge (*n* = 553)		394 (71.2)	
**Referred for nutrition support**			
Baseline (*n* = 2125)		172 (8.1)	
During hospitalization (*n* = 544)		102 (18.8)	
**Received nutrition support (*n* = 543)**		93 (17.1)	
**Reduced intake in hospital (*n* = 542)**		295 (54.5)	

Data are presented as *n* (%), mean ± SD (median) and ranges. * Significantly different compared to admission BMI (*p* < 0.001, paired values). ^1^ Reflects weight loss in the 6 months prior to admission. ^2^ Reflects weight loss during hospitalization. Abbreviations: NRS: nutrition risk screening; BMI: body mass index.

**Table 4:** Associations between at-risk for malnutrition on admission to hospital and variables.

	AT RISK OF MALNUTRITION	NOT AT RISK OF MALNUTRITION	*p*-values
	Mean ± SD, or %	Mean ± SD, or %	
Age (years) (*n* = 2123)	44.52 ± 16.28 (*n* = 1286)	45.41 ± 15.46 (*n* = 837)	*p* = 0.20 *
% Weight loss during hospitalization (*n* = 534)	2.11 ± 8.51 (*n* = 356)	0.77 ± 6.25 (*n* = 176)	*p* = 0.06 *
BMI on admission (kg/m^2^) (*n* = 2122)	23.27 ± 6.71 (*n* = 1287)	26.93 ± 7.09 (*n* = 835)	*p* < 0.001 *
BMI at discharge (kg/m^2^) (*n* = 534)	22.55 ± 6.69 (*n* = 356)	26.56 ± 7.97 (*n* = 176)	*p* < 0.001 *
In-hospital complications (no) (*n* = 534)	0.85 ± 1.22 (*n* = 356)	0.68 ± 1.11 (*n* = 176)	*p* = 0.11 *
Hospital length of stay (days) (*n* = 1985) ^1^	6.71 ± 5.62 (*n* = 1183)	5.71 ± 5.26 (*n* = 802)	*p* < 0.001 *
Complications in 3-months post-discharge period (no) (*n* = 198)	0.35 ± 0.58 (*n* = 117)	0.29 ± 0.55 (*n* = 81)	*p* = 0.21 *
Readmissions in 3-month post-discharge period (% yes) (*n* = 66)	72.7 (*n* = 48)	27.3 (*n* = 18)	*p* = 0.245, Chi^2^ = 1.35 **
Mortality (% yes) (*n* = 99)	81.8 (*n* = 81)	18.2 (*n* = 18)	*p* < 0.001 Chi^2^ = 21.68 **

Statistical analysis used: * ANOVA—analysis of variance. ** Maximum-likelihood (ML)-Chi square test. ^1^ The non-surviving patients were excluded. Abbreviations: BMI: body mass index.
